# Valedictory Address to the Graduating Class of the Baltimore College of Dental Surgery

**Published:** 1849-01

**Authors:** E. B. Gardette


### ARTICLE VI.

*Valedictory Address to the Graduating Class* of the Baltimore College of Dental Surgery. Delivered at the Invitation of the Faculty. By E. B. Gardette, M. D., March 1st, 1849.

*Gentlemen Graduates:*

It has become a custom in the institution, of which you have the good fortune this year to be the graduating class, to address you in the language of congratulation or advice, (imitating our peers of other colleges,) at the moment of your separation.

Having accepted the flattering invitation of your faculty to fulfil this duty, I bring to it, I fear, but meagre abilities for the performance of my task.

It was the advice of Cicero, that in writing an oration, a man should "begin in the middle and work out at both ends"—and if from my inexperience in such matters, I should, without designing it, pursue such a course, pray remember it is from one who perhaps has rather felt a pride in belonging to the plain practical class—a working man in contradistinction to a speaking one: I mean most especially with reference to our common profession.

You have listened this morning to a chaste, learned, and beautiful discourse, from the mind of a gentleman, and a classical scholar, which in truth leaves me nothing to say. But I honestly address you in a disposition to benefit you, and if success attend the effort in proportion to the strength and sincerity of this feeling, I

shall have no cause to regret that for once, on this first and only occasion, I depart from the quiet track of individual or solitary labor and usefulness.

I am to look upon you, gentlemen graduates, as dentists—not merely because you have earned diplomas from this college of dental surgery, but because I have witnessed the work from your hands, in various departments of the profession to which you aspire.

But you are dentists without practice, (most of you at least,) in the true sense of the term; and it is in reference to the nice connection between the knowledge of a profession or business, and the exercise of its duties for public benefit and advantage, as well as for individual prosperity, that I now propose to speak: for you will soon be called upon to test this question—the practical application of your knowledge—and will, possibly, derive some aid from the experience and observation of one who has passed through that ordeal.

It would often seem to be a single error of judgment among men, in their mode of interpreting the true affinity between preparation and practice, that determines the amount and character of their success in professional life: they feel the influence of their decision for good or evil, from the period at which, like yourselves, they start as young dentists, carving out a course of duty, until that doubtful time when the public shall have placed them in enviable elevation, or cast them amid the greater numbers, that live, and die unknown, and unregretted.

To succeed in practice, then, let us look, for a single moment, at the nature of our duties.

We are called upon to prevent or remedy diseases in the human teeth, those sensitive and precious organs so closely allied to health and comfort with man, and even more jealously watched and valued by woman.

The operations of the dentist are necessarily more or less painful, and the nervous timidity and reluctance with which he is generally approached, claim at his hands both sympathy and indulgence towards his patients. Whilst inflicting pain upon the body, it is both a duty and a blessed privilege, as far as practicable, to soothe and divert the mind. The anticipations or preconceived estimates of physical suffering, are at times so strong and vivid as to take the place of reality: the imagination measures pain before we have experienced it, and with a dogged tyrannical will she even assumes the high judgment seat of the sensorium.

Manipulations upon living organs of acute sensibility, therefore, call for the exercise of something more than mere mechanical dexterity; a nice discriminating judgment, good taste, a knowledge of characteristics and peculiarities in poor human nature, titled or appareled as it may be, whether in great or small specimens, will give an operator the power to mitigate unavoidable ills, through pleasant and engrossing mental associations.

I recall at this moment a graphic description of a dental operation from the lips of a gifted and sensitive friend, which embodies the thought I would convey so truly, that I beg to quote him from memory. Thus he spoke:

"With my head immovably secured against the chair back, my mouth stretched to its utmost, and every nerve in my frame in the attitude of resistance, he sawed away at one of my strong back teeth with the industry and monotony of a wood sawyer: no cessation or interruption, no word of comfort or inquiry, no remark or reflection to interest the mind; but it was one continued, undisturbed, horrible grating of file against bone and enamel, which became unendurable agony of body and thought. I could cheerfully have borne ten times the amount of actual pain in pleasanter company, and with something like sympathy or interest in the operator."

Supposing his preparatory knowledge and skill to be all that can be desired or acquired, the usefulness and success of a dentist will still depend somewhat upon the amount of interest and confidence he is able to inspire in those who consult him. And how shall he obtain this boon? how best accomplish so important a result. I answer—by establishing a high professional character.

And here let me say a few words as to the distinction I would draw between character and reputation.

The two terms (character and reputation) are commonly supposed to be synonymous, or at least are so used continually, an error by no means confined to our vocation. It is a common fault, whether we look to other professions and the reputations or characters that men have formed for themselves, or whether we turn to the same features of social life.

Their reputations for wealth, the fictitious capital of the merchant or banker (more cunning than wise) who gets extensive credits upon his reputation, until stoppage or failure brings investigation; his accounts are balanced, and he is found sadly deficient in assets to meet the just demands against him. And when executors to an estate come to close the last accounts, heirs as well as creditors too often discover the distinction between the reputation for wealth and its reality.

Your character is an actual and important part of yourself—your reputation merely what others choose to make out and determine.

There are reputations for learning, for wit, for greatness, many of which "loom large in the distance," like ships at sea; and hence, perhaps, it is said, "no man is a great man to his *valet de chambre*." Close examination of the real substance, the characters of men, will make any one of you at least as good a judge as the menial just named, of the distinction between truth, which is character, and its shadow, which is reputation.

Men who seek to make professional reputations merely as a means of profit, (and may I not say in reference to the number of such, that "their name is legion,") you will readily recognize in the endless devices by which, in one shape or another, appropriate to time and place, they are constantly aiming to be before the public eye. As *soi disant* dentists, they are found in remote time at the corners of the most frequented streets, with a display of instruments, studiously dressed, a mountebank with servant in attendance, not less remarkable than his employer for some outward indication of greatness, and both master and man equally busy in making reputation by deceiving the credulous and astonished crowd around them. Whilst the more or less dexterous charlatan mutilates the patient, his aid de camp (who may chance to be his brother in livery) is profitably engaged recommending and vending nostrums by way of reputation for both the devil and his drops.*

In more modern times we find that the facility of newspaper reputation answers his purpose better, and he has left the highways of large cities, to figure in the advertising columns of every public print in which he can manage to publish his name, his painless, wonderful cures and his moderate charges—and thus he makes reputation.

The *modus operandi* of these modern gentlemen is not so very different *in-doors* from the *out-door* practice of the ancient man of hooks and pelicans. To make reputation now a days, he rents a large and elegant house, far beyond his wants, and not unfrequently beyond his means; his internal arrangements are not only costly, but calculated to inspire the thought that he

*If an apology is needed for introducing his satanic majesty into such good company, even figuratively, it may be found perhaps in a reminiscence of my childhood.

The hall of my father's house, my early home, contained among other pictures, one of a quack dentist of Madrid; it was an ancient painting of the Spanish school at the beginning of the eighteenth century, representing a group of many figures as large as life. The scene was the street of the Spanish capital, the quack occupying the centre, was in the act of drawing a tooth from the wide stretched mouth of his customer: he was an old sinner, with horrible expression of countenance, and wore an endless number of decayed teeth strung around his neck, trophies probably of former butcheries. His servant was the next prominent figure, a tall sallow goggle-eyed creature, holding a basin and towel in front of the victim, and was also distinguishable from the herd of starers around, by a high chicken feather in his gaudy cap, and the fantastical labels about his advertising person.

The wretchedness and suffering expressed in the features of the poor woman, were irresistibly painful to behold, and the expectant whose turn came next, was no less plainly indicated by his palm upon his cheek. But in the picture at least his turn never came, for there the agony of the pitiable female then being tortured was as permanent as the painter's canvass.

To my childish eye and fancy, this big picture was dreadful, and the faces of the old quack and his queer aid de camp, remain as lasting impressions which I can now compare to nothing but satan and one of his imps.

who has provided them possesses a knowledge of the sciences, or has some favorite pursuit as one of their votaries. For we are told that in the great cities of London and Paris, you find in his reception rooms, most valuable collections of ornithology, concology, or numismatics, whilst their illiterate owner would be unable to explain the simplest feature of the science they refer to, and has had no part in the merit of their selection or classification.

You may recognize this eagerness for undeserved fame and success in almost every act of his life, and particularly at the beginning of his career: in his readiness to adopt all new experiments that may seem to promise business or popularity, in accommodating his practice to the dictation of each patient, in the eager proffer of his services, unsought and gratuitously, to gentlemen in other professions, and especially to medical men and the clergy; a course which may be highly proper when dictated by personal regard or benevolence, or when professional services can be reciprocated.

Magnificent instruments, the handles adorned with pearl or ruby, not designed for use, nor particularly adapted in form or temper to the business of any operation, constitute another in-door device to make reputation; or, as one of these mushroom dentists has expressed himself, "to *take* with the southern people."

But I willingly turn from this painful picture to its opposite; to the man who seeks with all his talents, his industry and skill, to establish a high professional character, and only cares for that reputation which is derived from, and belongs to, a good character.

He conscientiously prepares himself with a correct knowledge of the principles that govern his business; his arrangements for its practice are modest and consistent, he seeks to make friends by his good conduct, his courteous manners and correct habits; he aims to establish a good character in his profession and out of it, by an upright, frank, and manly course. His patients are sure to get the benefit of his best judgment in reference to each trouble about which he is consulted, and his honest hand will perform its duty with gentleness and ability. His advice and his work will remain, the one in the memory and the other in the mouth, as testimonials of good character; and each professional visitor will voluntarily aid in extending for him a well founded and enduring fame.

You will see him steadily at his post, with just pride and anxiety for the constant improvement of his operations; doing justice to the confidence reposed in him, by earnest efforts to relieve suffering and remedy disease, while at the same time he claims consideration and respect for his professional opinions and labors consistent with a true sense of their importance.

The reputation a man may create for himself by empty show and doubtful promises, is never so enduring as that fame which others are forced to extend to him as the unavoidable result of his own good deeds. The one is a credit for abilities which he may not possess, whilst the other is simple and just interest or return for capital advanced in the substantial and valuable form of benefits, conferred upon the community amidst whom he exercises his talents.

Thus in the distinction I have attempted to draw between character and reputation, have I not successfully pointed to the true mode of inspiring confidence, that great element of professional success, prosperity and usefulness. A man without a good character, may, it is true, for a brief space, have a good reputation; but with a good character he is not only more sure of better reputation, but he will have also the precious inward comfort of knowing that it is deserved.

I need not ask you which line of conduct you are disposed to pursue; the one having for its object the formation and establishment of high professional character, or the other leading to a weak ephemeral, money-making reputation. Your good sense has no doubt determined in favor of the right course, and therefore permit me to remind you, that this narrow path is the difficult one; it will claim at your hands the exercise of much patience, and bring you, perhaps, neglect and injustice at the commencement of your careers: it will often demand sacrifices, and the courageous practice of self-denial. But in the end, you will thus gain the inestimable reward of self-approbation, and the lasting respect and gratitude of all worthy acquaintances you may form in your path through life.

The general standing of the profession itself, of which you are now individual members, must depend greatly upon the characters of the men who represent it; and hence you have an additional motive to render yourselves worthy of the highest regard. Expect to borrow nothing from the reputation of your profession, but to lend it importance, dignity and value, by bringing to it the influences of your own good names. You will thus triumph over those narrow and prejudiced minds, still to be found in civilized intellectual communities, who will judge you, not by your merits or your character, but by the grade or standing of your occupation, according to their estimate of it, or some such small medium of judgment, suggested by their own weak pride or false pretensions. Let a strong sense of duty and a just discharge of it, prove your value to the world, and you may then answer the illiberal and misjudging, in the words of the sage, who when informed of the dislike and injustice of his neighbor, said, "I will treat him so well and become so worthy of his respect, that he shall be compelled to esteem me."

All vocations or pursuits in life have their objectionable and agreeable characteristics—their shade and their sunshine, and it would be, no doubt, a more easy than profitable task, to point out the *ratio* belonging to that of the dentist, whether of pain or gratification. He that selects it for himself, must resolve to pursue it honorably and usefully, and find consolation in its pleasant features for the admixture of the painful or disagreeable.

I am disposed however to notice here, one great and just cause of complaint, and which in early life has been a sore trial to most of us; I do so the more willingly, as I may chance to convey, with due deference, a gentle lesson to some other listener besides the young gentlemen graduates, to whom I more particularly address myself.

I refer to the desire or practice of dictating to the dentist, rather than seeking his advice; going to a professional man as you would to a tinker or a laborer, and expecting to hire his hands to do that which is not sanctioned by his judgment. A man of intellect who has knowledge and experience in his particular vocation, is thus reduced to the level of a mere machine; he is bought, set in motion, and directed by the caprice and the money of his employer.

Our great Franklin has said, that "if you hire an individual to do a piece of work of which he disapproves, you hire in truth but one-third of a man; his head and heart are against you, and only his hands are for you." The illustration applies equally to professional pursuits as to common labor, and Franklin's third of a man, I grieve to say it, is too often to be seen in the intercourse between patient and dentist.

It has been less difficult for me to point out the duties of our profession to suffering humanity, than to determine upon what is due from the public to the dentist; and expressing some views on that subject not long since to a distinguished gentleman, philanthropically interested in the question, he urged that they might be my theme on this occasion. Diffident and poorly qualified as I may be to satisfy his expectations, I am not willing entirely to disregard the wish of one who so deservedly holds a high station in this institution.

The relative claims of the dentist upon the community, not less than those of all professional men who do their duty, have not been properly regarded or duly estimated in this country by the majority of those who need and seek their services; and although I do not forget that it amounts to a common adage, that every man thinks his own trade the most slavish, and the most abused by the world, we may be allowed the universal privilege of complaining a little.

A distinguished divine, formerly of Philadelphia, whose extensive usefulness brought him in contact with very many people, placed a sign upon the outside door leading to his study, with these words—"*be brief, time is short.*" I have heard, too, of a popular dentist in one of the great cities, whose ante-rooms contain printed directions, or little instruction books, how to behave oneself in his *sanctum sanctorum.* But without pretending to advocate, for an instant, what may seem an assumption of consequence in the latter, I can but think that the busy dentist may, with some propriety, imitate the example of the reverend gentleman, and remind his visiters, if necessary, that time lost is suffering to the patient in the chair, and a tax upon him who officiates.

There is an immediate and unavoidable connection in this subject with the social position of the dentist, and it is the general character of his situation, to which I beg to be understood as referring exclusively.

The well bred and well educated, are only a very small proportion of those who require the services of the dentist; his doors are open equally to such as neither know how to appreciate him nor the nature of his operations. He is ready to relieve all kinds and classes of persons, and he encounters them too in moods and tempers not *very* particularly calculated to promote pleasant or polite intercourse.

> "There was never yet philosopher
> That could endure the tooth-ache patiently."

A strong sense of his duty, and the influences of benevolent sympathy, not less than the laws of hospitality (for the dentist is at his home) remind him to bear submissively the assaults upon his good nature and selfrespect. But patience has its limits, even in the dentist, and when a *fond parent* return to his office with a refractory child the fourth or fifth time, to have the same insignificant operation performed, and which, though requiring but a moment's time, has already wasted five or six hours in as many succeeding days, is it *very* wonderful, I ask, if the dentist ceases to feel much solicitude to relieve, or interest to serve the patient.

The preparations or educating of mind and feelings for enduring operations upon the teeth, is no part of the study of dental surgery, or of a dentist's duties, and parents mistake, we think, both their own and his position, when they expect all the persuading—the making up of the mind (to use the familiar phrase)—to take place in the operating chair.

The complaints in these respects, however, relating to the young and the extraction of teeth, are of small importance and the errors very excusable, when compared with the annoyances that "*grown up children*" too often inflict upon men, whom, be it remembered, they have voluntarily selected and applied to as their dentists; and I will only touch upon some of these, that you may be the better prepared to meet them in your approaching offers of usefulness to the people among whom you may practice.

You will be told, in no measured or cautious phraseology, that your profession has done more harm than good; that dentists have no feeling; that they seem to enjoy the infliction of pain upon others; and more often still you will be urged to give no *unnecessary* pain, as though it was entirely optional with yourself. Should you chance to be overrun with occupation, (the good fortune I sincerely wish you) and your time necessarily engaged for some days in advance by those kind and considerate ones who in patient confidence are waiting your convenience, you will still be importuned by late comers, to give them the hours which are no longer at your disposal; not yielding to these entreaties, you will be called disobliging, cross, presuming, or perchance you may only perceive in the cold *hauteur* of some wealthy west-end republican, that you have given great offence.

Your professional advice will be sought, "*merely to know your opinion*" as to what is the best remedy in the case, and what the consequences of such and such a course: and these consultations would seem flattering to your standing and your judgment as a dentist. But in many instances this compliment is all the advantage you are to derive from your visitor, who from your office goes immediately perhaps to that of a more universal man, the barber tooth-drawer and chiropodist, and for the moderate sum of a few shillings, the operation you recommended is performed to the patient's satisfaction.

With your hands and your head full of business not to be satisfied or disposed of short of a fortnight's assiduous labor, you will have unexpected demands upon your time from persons who have "*just made up their minds,*" and brought up their courage, to endure various operations; the one to have any number of teeth and fangs removed, and an entire set of artificial teeth of their own selecting at once substituted: others, to have performed that most indefinite and terribly complex duty to a dentist, which they so graphically express by—"*I want you to fix all my teeth to-day, sir.*"

You will, a few years hence, without doubt, realize better than you can at present, how singularly unreasonable such propositions, soberly made, are to the dentist who is fully occupied: and he, of all others, is the one most likely to receive such applications, from persons who seem to forget that there must be two parties to every agreement, as well as "two sides to every question," and that "*qui compte saus son hote, compte deux fois.*" Whether coming from a distance or residing in the same town with you, they make their arrangements and calculations without consulting you, as to what is to be done, how it is to be done, and when it must be accomplished.

I spoke a moment since of influences connected with home and hospitality, and these terms remind me of some features of distinction between the professional life of the doctor and the dentist, which I beg to notice.

The visits of the physician being chiefly and necessarily at the houses of his patients, call forth the better feelings of the heart, arising from a grateful sense of his value; he is in a degree, both a guest and a benefactor. The rooms are dusted and put to rights, the little ones are scrubbed, and clean aprons at least made to cover the spots beneath, before the time appointed for the doctor's daily call; and when any of these evidences of respect are wanting on the physician's arrival, the apology for their absence is not forgotten even by the humble and uneducated.

Pondering jealously sometimes upon these things, and with envious eye gazing through the window of his prison-room at the medical man of his own age, as his handsome two horse easy-hung cab drives dashingly by, the dentist might doubt whether the established custom of receiving rather than paying visits, has been to him a wise arrangement. Deprived of wholesome air to an injurious extent, and of the cheering influences upon health of body and mind derivable from change of scene and moving amidst the world of events and interests presented by a populous city, the dentist vainly claims sympathy for his close confinement, his sedentary habits, and the "wear and tear" upon his frame and depressed spirits. A few are found, it is true, whose robust health and elasticity of mind sustain them against such gnawings at the springs of life for a goodly number of years, but as a general rule, we believe the career of the dentist has been short, and its end melancholy.

The medical man may be said to hold his position in every family where his professional services have, year after year, brought him in contact with its members under their own roof, and I am far from disputing his rightful claims to such consoling notice. But the question has forced itself upon my mind, whether the intercourse between patient and dentist, if existing under the same circumstances, would not have placed him upon a similar footing with the family physician and surgeon. I merely throw out the suggestion here, without time to examine properly so nice and important a question in all its bearings upon the present system, which we know possesses very great advantages and recommendations.

The delicate subject of professional fees or compensation, is still open before me, as a broad endless page upon which to "score up" loud complaints; just and well founded exceptions to the small penny-wise policy of those who seek and those who offer cheap dentistry. Aye, who not content with merely seeking it for themselves, recommend it strongly to friends and acquaintances, and apply the gentle terms of "exorbitant" or "unreasonable impositions" to all other kinds. The liberal patrons of our science, make bargains in advance for operations to be performed; they desire the dentist to undertake them "by the job," to "lump it," as they would hire a carter to remove rubbish, and put their own valuation upon the time and talents of him whom they honor with their preference.

There is too much to be said upon this branch of the evils that surround the practice of your profession, but many of which, having been remedied or regulated in some degree by your predecessors, you may be fortunate enough to escape: and yet I would not have my unwillingness to enter more minutely into the subject, for a single moment, attributed to a want of data to go upon, in an attempt to prove (time and opportunity being appropriate) that the well informed and faithful dentist who does his duty and whose fees are uniform, is not justly open to the charge so commonly made against him in the "hue and cry" of "extortion!" "extravagance," &c. &c.

The estimate of professional services, whether in the medical man, the surgeon, the legal adviser, (the lawyers know how to take care of themselves,) or the dentist, is surely not to be determined by each party that seeks him for aid or relief; if so, the standard of value would change often and singularly, varying according to the amount of liberality or gratitude; and the ability to give the "*quid pro quo*," would, we fear, rarely govern in settling the just pecuniary obligation for benefits conferred.

The views I have thus plainly expressed may possibly seem strange and almost incredible to you, young gentlemen, but they are, not the less, true delineations, taken from the book of experience: believe me, I have no desire to alarm you with an overdrawn picture of professional difficulties and annoyances, many of which we would fain hope originate only in the paltry characters of men, styling themselves dentists, and who have been trusted to the great disadvantage and sad regret of those by whom they have been employed.

But "there is yet balm in Gilead," and possibly I may assist you in discovering where it may be found.

Heaven, in dividing its gifts of good and evil on earth, has allotted, it would seem, an undue share of suffering to woman, of whose delicate organs you will have the care. Her teeth afflictions and her gratitude for relief from them, make her especially your friend: in her bright smiles and warm words of praise, you will find compensation for the indifference or injustice of men.*

* It may not be considered out of place to relate here a professional anecdote of my father, amply illustrating what I have just expressed.

Some of you have perhaps sought accommodations as travellers at the "Mansion House" South Third street, Philadelphia, which was for many years so admirably kept by Mr. Joseph Head. That elegant building was originally the residence of William Bingham, Esq., an English gentleman of great wealth, and who boasted the possession of a magnet more precious than his mansion or his money, in the person of his most accomplished, excellent and beautiful wife. Mrs. Bing-

The human tongue is a great exaggerator, and proverbs have given wide-spread fame to that of woman in particular; but whether this be just or otherwise, there is certainly one sense in which the "unruly member" of both sexes will exaggerate good or evil, and that is in defence or examination of its own limited domain, the mouth. Within these narrow archways, a rough corner or sharp ragged edge, which to the eye seems as nothing, is to the delicate, searching and sensitive tongue, a serious inroad and most aggravating annoyance.

Hence the work of the dentist brings him, it may be said, immediately "under the tongue of good or evil report," and that too most frequently in the head of woman. The association and responsibility is one to be proud of; as much to be dreaded in its displeasure, ham, about the year 1788 or 89, a sensitive, timid person, who had been afflicted for a considerable time with inflammatory tooth-ache, was unable to obtain relief. She could not command the nerve necessary to bear extraction of the tooth, and yet was induced by her friends and physician to send for a young French dentist, then recently arrived in the city of Penn. A first, a second, and a third visit, in three successive days, were paid in vain; the patient was weakened and nervous, while the dentist was polite and persuasive. But Mrs. Bingham, in her courteous apologies for causing such fruitless visits, insisted that her resolution held good until she saw the dentist, but with the sight of him her courage all fled. As a little "*ruse de guerre*," the operator suggested that when her next resolve was taken, she should be blindfolded, then send for him and on his approach, without a single word, open her mouth. This plan was adopted with success, and whether from the small amount of pain, compared with heightened fears and expectations, or from the actual skill of the dentist, it matters not—the lady was relieved, delighted and happy; she was grateful and generous, and with the kindest expression of her thanks, she pressed into the operator's hand as they parted, a little silken purse. On examining his fee, unclaimed and most unexpected, he found it to be *fifty guineas*:—but he had acquired that which proved more valuable to him than a thousand such; a warm friend in a lady of rank, one who lent to, rather than derived it from, the great fortune and position she enjoyed.

If I might be permitted to give a name to my little family story, I should call it, "the *poetry* of *tooth pulling*."

as it is to be blessed and relied upon in its movements of grateful approbation, for this is the "Balm in Gilead." Let your care and your skilful manipulations respect its province: let your gentleness and good usage cultivate its friendly and warm interest: let your merits and estimable qualities of mind and heart deserve its kind and encouraging notice; and when you have done these things, when you have justly earned the respect and gratitude of refined woman's tongue, you have established your good characters, your fortunes are secure.

It only remains for me, in the name of the faculty, to thank this good audience, and offer you, gentlemen graduates, our best wishes and parting salutations.

